# Correlation and Significance of Urinary Soluble Fas and Vascular Endothelial Growth Factor in Bladder Urothelial Cancer

**DOI:** 10.1155/2015/383509

**Published:** 2015-12-20

**Authors:** Huixiang Yang, Zhiyong Wang, Yong Guo, Zemin Wang

**Affiliations:** ^1^Department of Andrology, First Hospital of Shijiazhuang, Shijiazhuang 050011, China; ^2^Department of Urology, The First Hospital of Shijiazhuang, Shijiazhuang 050011, China; ^3^Department of Urology, Affiliated Hospital to Chengde Medical College, Chengde 067000, China

## Abstract

*Aim*. To investigate the correlation and significance between the urine soluble Fas (sFas) and vascular endothelial growth factor (VEGF) expression in patients with urothelial bladder carcinoma (UC).* Methods*. The level of sFas was measured by enzyme-linked immunosorbent assay (ELISA) and the expression of VEGF protein in UC surgical specimens was screened by immunohistochemical method. These data were analyzed through SPSS 13.0 software.* Results*. The urinary sFas levels were significantly higher in the patients with UC than in those without cancer (168.0 ng/mL ± 84.6 versus 56.2 ng/mL ± 37.0; *P* < 0.05) and in the cases with a higher stage or grade than in those with a lower stage or grade (each *P* < 0.05). They had a positive relationship between the expression of VEGF protein and the pathological stage or grade in UC tissues (each *P* < 0.05). Spearman rank correlation test showed a significant correlation between sFas levels and VEGF expressions (*R* = 0.882, *P* < 0.05).* Conclusions*. The effects of sFas and VEGF may play important roles together in the occurrence and progression of UC. Joint detection of urine sFas plus VEGF protein may provide valuable solutions to improve the diagnosis and treatment of UC.

## 1. Introduction

Urothelial bladder carcinoma (UC) is the most common malignancy of the urinary tract in China. The cancer has the highest recurrence rate of any solid tumor. Unfortunately, the recurrence and progression of the disease may not be well predicted in individual patient using the clinical and pathological parameters including tumor size, grade, stage, the multiplicity of lesions, and prior recurrence rate [1]. Therefore, it is critical to identify new biochemical factors. Obviously, determining the potential tumor markers of UC can indicate the different risks of recurrence and progression in patients; it even provides a targeted diagnosis and treatment for the patients. And tumor markers subsequently help to design the individualized therapeutic strategy, which will greatly improve the survival time and quality of life of patient with UC. At present, however, none of tumor markers for urothelial carcinoma have been applied to clinical practice alone and proved by multicenter and large scale study.

Previous studies have shown that apoptosis plays a very important role in the occurrence and development of bladder cancer [2], which provides a new way for diagnosis and treatment of UC. For apoptosis, the Fas/Fas ligand (FasL) system is a key regulator. Fas is a transmembrane cell surface receptor that triggers cell death upon binding to FasL, which is an anchored cell membrane protein exposed to the extracellular space [3]. Fas can be found in two forms, the transmembrane form and the soluble form. Soluble Fas (sFas) can bind to membrane-bound FasL, thus blocking the binding of the ligand to the Fas receptor, preventing apoptosis induction in the target cell, and enhancing the immunosuppressive effects of tumors. Some research shows that sFas can be detected in the urine or serum of the bladder cancer patients. We [4, 5] and Svatek et al. [6] noted that the urinary soluble Fas levels in patients undergoing surveillance for urothelial carcinoma were higher in cancer patients than the control group and that the urinary sFas was an independent predictor of bladder cancer recurrence and invasiveness in patients who had a past history of UC.

Interestingly, vascular endothelial growth factor (VEGF) can inhibit apoptosis [7]. VEGF, which is considered the most important of the angiogenic stimulators during tumor angiogenesis, has been implicated as a major survival (antiapoptotic) factor [8]. Angiogenesis, the growth of new blood vessels from the existing ones, is a marker of aggressiveness, which is known to play a leading role in the survival, proliferation, and metastatic potential of malignant tumors [8, 9]. It is a normal physiological process in fetal development and wound healing. But, in cancer, it is essential for tumor growth and metastatic spread. VEGF is expressed in bladder tumors, and the increased expression of VEGF is associated with higher tumor stage and progression [8–10]. However, to the best of our knowledge, no studies have applied combined detection of urinary soluble Fas and VEGF in UC.

In this study, we investigated the correlation and significance between the urine soluble Fas and vascular endothelial growth factor expression in patients with UC.

## 2. Methods

### 2.1. Patients

In this study, patients suffering from UC were recruited at random between January 2008 and May 2013 at the First Hospital of Shijiazhuang (Shijiazhuang, China) and the Affiliated Hospital to Chengde Medical College (Chengde, China). The classification of tumor stage and grade was made according to both 2002 TMN classification and World Health Organization (WHO) criteria (2004 version). None of the patients underwent preoperative adjuvant chemotherapy or radiotherapy. Cancer-free controls, without a history or family history of cancer or other genetic diseases, frequency matched by gender and age (±3 years), were recruited from individuals who visited the same hospitals. Subjects who suffered from renal insufficiency, heart failure, upper urinary tract tumors, intraoperative vesical perforation, or significant hematuria were excluded. We analyzed 82 patients (51 males and 31 females) with UC and their mean age was 58.8 years (range: 21–84). Of the 82 urothelial bladder carcinoma cases, with size from 0.2 to 4 cm in diameter, 56 were histologically diagnosed as nonmuscle invasive bladder cancers (NMIBC), 26 were muscle invasive bladder cancers (MIBC), 42 were low grade papillary urothelial cancers (LGPUC), and 46 were high grade papillary urothelial cancers (HGPUC). The control cohort consisted of 82 cancer-free individuals including 15 with benign prostatic hyperplasia, 17 with stones, 20 with nonspecific urinary tract infections, and 30 healthy volunteers.

The study was approved by the Ethical Review Committee of First Hospital of Shijiazhuang and conformed to ethical guidelines of 1964 Declaration of Helsinki. All UC patients and cancer-free controls provided written informed consent before enrollment.

### 2.2. Enzyme-Linked Immunosorbent Assay

The first morning voided urine sample (10–20 mL) was obtained for measurement of sFas from preoperative patients and controls. Urine samples were centrifuged immediately at 2,000 g for 10 min and then frozen at −70°C until assayed. Levels of sFas in urine samples were quantified by a commercially available enzyme-linked immunosorbent assay (ELISA) kit (R&D Systems Inc., USA) according to the manufacturer's protocol and expressed as ng/mL. Every sample was measured 3 times and the mean was calculated for data analysis.

### 2.3. Immunohistochemical Study and Scoring

The immunohistochemical (IHC) study was carried out on formalin-fixed, paraffin-embedded tissues. Serial sections of the tumor tissues were obtained from archived paraffin-embedded tissue blocks. In all cases, the primary pathological diagnosis was confirmed by hematoxylin and eosin (HE) staining; then slides were stained for VEGF. In brief, all slides were deparaffinized in xylene and then rehydrated in ethanol. Subsequently, all sections were treated with 3% hydrogen peroxide for 5 min and heated in a citrate buffer using a steam cooker and then were incubated with the polyclonal primary antibody (VEGF, Boster Biological Technology, Ltd., China; 1 : 100 dilution) at 37°C for 2 hours. Secondary antibody and streptavidin-biotin complex (Boster Biological Technology, Ltd., China) were then applied at 37°C for 20 min, respectively. After rinsing, the slides were stained with diaminobenzidine as chromogen and counterstained with routine hematoxylin.

We used a semiquantitative analysis system to determine the VEGF immunostaining score according to the percentage and intensity of positive cells among less than 500 cells [9]. For percentage, 0~4 scores represent <5%, 5~25%, 26~50%, 51~75%, and >75% of the labeled cells, respectively. For the intensity, 0~3 scores indicate none, weak, middle, and strong staining, respectively. Multiplication of both scores decided the final score (VEGF score), ranging within 0~12. A double-blind analysis was performed by two independent pathologists. Each sample was scored twice. If the final scores had a more than 3-point discrepancy, a second evaluation would be performed. Using −~+++, VEGF score = 0, 4 > VEGF score > 0, 8 > VEGF score ≥ 4, and VEGF score ≥ 8, respectively.

### 2.4. Statistical Analysis

Clinical and pathological data were gathered from each patient. Patient data were analyzed, and descriptive statistics were used to summarize the study population characteristics. Statistical analyses were performed using SPSS 13.0 software (SPSS Inc., USA). The data of urinary soluble Fas level were presented as median ± interquartile range (M ± IQR). Differences in continuous variables were tested by Mann-Whitney *U* test. In order to derive the most appropriate sFas cutoff value for use in diagnosing UC, a receiver operating characteristic (ROC) curve was constructed. The value that maximized the difference between sensitivity and the false positive rate (Youden index = sensitivity + specificity − 1) was selected as the cutoff value.

The correlations of variables were evaluated with Spearman rank correlation test. All statistical analyses were two-sided with *P* value of less than 0.05 defined as statistically significant difference.

## 3. Results

**Figure 1:**
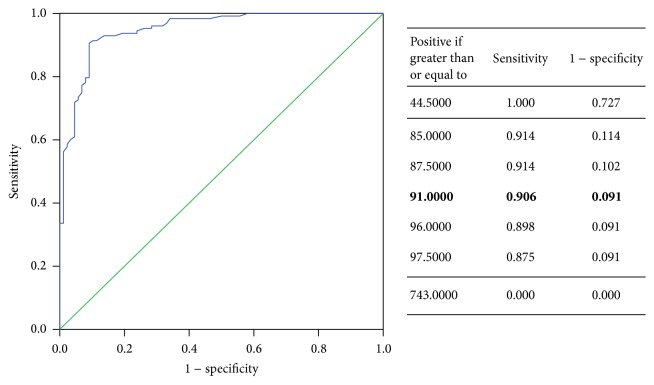
ROC curve of urine levels of sFas in the diagnosis of UC patients.

The ELISA technique was employed to determine urinary sFas concentration; the intra-assay coefficient of variation was 6.09%. The Mann-Whitney *U* test results showed that sFas levels were significantly higher in the urine of UC patients than in those without cancer (168.0 ng/mL ± 84.6 versus 56.2 ng/mL ± 37.0; *U* = 333.5, *P* = 0.000). On the basis of ROC curve, the area under the curve (AUC) was 0.928 (95% CI 0.887–0.969; Figure 1). By ELISA, the sensitivity and specificity of sFas for the patients of UC were 85.3% and 88.2%, respectively (*χ* ^2^ = 73.593; *P* = 0.000), if the cutoff value of sFas was selected as 91.0 ng/mL according to Youden index (Figure 1).

The association between the clinicopathological characteristics and urinary sFas levels and VEGF expressions is shown in Table 1. By Mann-Whitney *U* test, the urinary sFas level was significantly higher in cases with a higher stage or grade than in those with a lower stage or grade (each *P* < 0.05), while it did not differ depending on gender or age (each *P* > 0.05).

**Figure 2:**
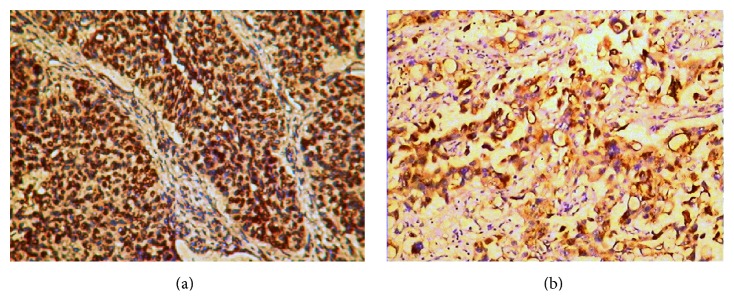
Representative examples of VEGF in UC. Positive VEGF immunoreactivity is detected in the membrane or cytoplasm of cancer cells. Original magnifications: ×40 (a) and ×100 (b).

Positive expression of VEGF was membrane staining or strong cytoplasmic reactivity (Figure 2). In 82 cases, the number of positive expressions of VEGF was 61 (74.4%). By the method of Spearman rank correlation test, they had a positive relationship between the expression of VEGF protein and the pathological stage or grade (each *P* < 0.05), so the positive rate of VEGF increased gradually with the progression of the pathological stage or histological grade of bladder cancer (Table 2). Compared with the VEGF negative expression group, the level of sFas was elevated in the VEGF positive expression group (*P* = 0.000, Table 1). And then the Spearman rank correlation test showed a significant correlation between the urinary sFas levels and the VEGF expression (*R* = 0.882, *P* = 0.000, Table 3).

## 4. Discussion

In recent years, the research based on Fas/FasL system has become one of the important advances in the field of molecular biology [11, 12], and higher sFas levels in the serum have been investigated in various cancer types [4, 13, 14]. Mizutani et al. [15] reported elevated sFas levels in the serum of patients with bladder cancer and suggested an association of elevated sFas level with poor prognosis in these patients. Conversely, Perabo et al. [16] argued that serum sFas seemed useless as a tumor marker and that it had limited relevance in laboratory investigations of bladder cancer. There are few data currently available in the literature concerning urinary sFas in bladder cancer patients [5–7, 17]. In the present study, we found that urinary sFas levels in UC patients were higher in cancer patients than the control group. Compared with current test, the detection of urine soluble Fas by ELISA has its advantages because of a higher sensitivity and specificity and lower cost. Then, according to our results, sFas continued to rise with the increase of the aggressiveness of bladder cancer, and it may be used as an indicator of the malignant degree of bladder cancer. We speculate that high-level expression of sFas protein in UC may be involved in the pathogenesis and progression of the disease, leading to the initiation of cell apoptosis and causing bladder cancer cells insensitivity to postoperative intravesical instillation and therefore more likely causing tumor recurrence. However, the exact mechanism remains unclear.

There are four main forms of VEGF, each one with a variety of functions, including recruitment and mitogenic stimulation of endothelial cells [18]. Level of VEGF has been shown to influence recurrence and survival in urothelial carcinoma [9, 10]. According to the results of this study, VEGF expression can be detected in both invasive and noninvasive disease, and the increased expression of VEGF was associated with increasing tumor stage or grade of bladder cancer, which means VEGF plays an important role in the progression of bladder cancer and its expression may serve as an important prognostic indicator.

Furthermore, we found for the first time that the levels of sFas were elevated with the increase of the expression of VEGF, so the effects of sFas inhibiting the apoptosis and VEGF activating angiogenesis have a close relationship with the occurrence and progression of UC. Soluble Fas and VEGF may play important roles together in the occurrence and progression of urothelial bladder cancer and interact with each other via signal transduction network and then influence biological behavior of the tumor, but the specific mechanism needs further research. This study also suggests that joint detection of urine sFas plus VEGF protein has a practical value for evaluating progression and prognosis of UC, which will provide valuable solutions to improve the diagnosis and treatment of bladder cancer.

In terms of limitations, since this study was a preliminary investigation, the number of patients was relatively small. We reckon the statement that soluble Fas level is higher in the high grade patients which was premature unless cases with comparable mass and different grade were compared. In the meantime, the origin of sFas in urine is unknown even though this has no visible effect on the final evaluation.

In conclusion, the results showed that urinary sFas levels in UC patients were higher in cancer patients than the control group. In the meantime, VEGF expression can be detected in both invasive and noninvasive disease, and the increased expression of VEGF is associated with increasing tumor stage or grade of bladder cancer. Furthermore, we found that the level of sFas was elevated with the increase of the expression of VEGF, so the effects of sFas and VEGF may play important roles together in the occurrence and progression of urothelial bladder cancer. This study also suggests that joint detection of urine sFas plus VEGF protein will provide valuable solutions to improve the diagnosis and treatment of bladder cancer.

## Figures and Tables

**Table 1:** Clinicopathological characteristics and urinary sFas levels and VEGF expressions of the UC patients.

Variables	*n*	Urinary sFas (ng/mL)	*P* value
		M ± IQR	
Gender			0.312
Male	51	177.3 ± 94.2	
Female	31	158.0 ± 68.8	
Age (years)			0.741
≤60	62	168.1 ± 72.0	
>60	20	162.5 ± 86.0	
Grade			0.039
LGPUC	42	157.0 ± 78.3	
HGPUC	40	179.6 ± 91.2	
T stage			0.000
NMIBC	56	139.0 ± 71.3	
MIBC	26	230.5 ± 113.2	
VEGF expression			0.000
Negative	21	92.5 ± 41.3	
Positive	61	194.0 ± 99.5	

NMIBC: nonmuscle invasive bladder cancer; MIBC: muscle invasive bladder cancer; LGPUC: low grade papillary urothelial cancer; HGPUC: high grade papillary urothelial cancer.

**Table 2:** The correlation of VEGF expression and the pathological stage and grade in the UC patients.

	VEGF expression (*n*)				*R* value	*P* value
	−	+	++	+++		
Grade					0.735	0.001
LGPUC	14	4	14	10		
HGPUC	7	8	9	16		
T stage					0.823	0.001
NMIBC	19	6	20	11		
MIBC	2	6	3	15		

NMIBC: nonmuscle invasive bladder cancer; MIBC: muscle invasive bladder cancer; LGPUC: low grade papillary urothelial cancer; HGPUC: high grade papillary urothelial cancer.

**Table 3:** Urinary sFas value in different VEGF groups of the UC patients.

VEGF expression	*n*	Urinary sFas (ng/mL)	*R* value	*P* value
		M ± IQR		
−	21	95.0 ± 36.5	0.882	0.000
+	12	129.5 ± 49.5		
++	23	158.5 ± 82.8		
+++	26	380.5 ± 327.5		

## References

[1] Cheng L., Zhang S., MacLennan G. T., Williamson S. R., Lopez-Beltran A., Montironi R. (2011). Bladder cancer: translating molecular genetic insights into clinical practice. *Human Pathology*.

[2] O'Kane H. F., Watson C. J., Johnston S. R. (2006). Targeting death receptors in bladder, prostate and renal cancer. *The Journal of Urology*.

[3] Ulukaya E., Acilan C., Yilmaz M. (2010). sFas levels increase in response to cisplatin-based chemotherapy in lung cancer patients. *Cell Biochemistry and Function*.

[4] Yang H., Li H., Wang Z., Gao J., Guo Y. (2013). Is urinary soluble Fas an independent predictor of non-muscle-invasive bladder cancer? A prospective chart study. *Urologia Internationalis*.

[5] Yang H., Gao J., Wang Z., Zhai X. (2013). Detection of soluble Fas in urine for the diagnosis of bladder cancer. *China Journal of Modern Medicine*.

[6] Svatek R. S., Herman M. P., Lotan Y. (2006). Soluble Fas—a promising novel urinary marker for the detection of recurrent superficial bladder cancer. *Cancer*.

[7] Ji Y., Chen S., Li K., Xiao X., Xu T., Zheng S. (2014). Upregulated autocrine vascular endothelial growth factor (VEGF)/VEGF receptor-2 loop prevents apoptosis in haemangioma-derived endothelial cells. *The British Journal of Dermatology*.

[8] Verma A., Degrado J., Hittelman A. B., Wheeler M. A., Kaimakliotis H. Z., Weiss R. M. (2011). Effect of mitomycin C on concentrations of vascular endothelial growth factor and its receptors in bladder cancer cells and in bladders of rats intravesically instilled with mitomycin C. *BJU International*.

[9] Chen J.-X., Deng N., Chen X. (2012). A novel molecular grading model: combination of Ki67 and VEGF in predicting tumor recurrence and progression in non-invasive urothelial bladder cancer. *Asian Pacific Journal of Cancer Prevention*.

[10] Kopparapu P. K., Boorjian S. A., Robinson B. D. (2013). Expression of VEGF and its receptors VEGFR1/VEGFR2 is associated with invasiveness of bladder cancer. *Anticancer Research*.

[11] Guo C.-L., Yang X.-H., Cheng W. (2014). Expression of Fas/FasL in CD8^+^ T and CD3^+^ Foxp3^+^ Treg cells—relationship with apoptosis of circulating CD8^+^ T cells in hepatocellular carcinoma patients. *Asian Pacific Journal of Cancer Prevention*.

[12] Dai X. L., Zhou S. L., Qiu J., Liu Y. F., Hua H. (2012). Correlated expression of Fas, NF-*κ*B, and VEGF-C in infiltrating ductal carcinoma of the breast. *European Journal of Gynaecological Oncology*.

[13] Codony-Servat J., Garcia-Albeniz X., Pericay C. (2013). Soluble FAS in the prediction of benefit from cetuximab and irinotecan for patients with advanced colorectal cancer. *Medical Oncology*.

[14] Boroumand-Noughabi S., Sima H. R., Ghaffarzadehgan K. (2010). Soluble Fas might serve as a diagnostic tool for gastric adenocarcinoma. *BMC Cancer*.

[15] Mizutani Y., Yoshida O., Bonavida B. (1998). Prognostic significance of soluble Fas in the serum of patients with bladder cancer. *The Journal of Urology*.

[16] Perabo F. G. E., Mattes R. H., Wirger A. (2001). Soluble Fas and Fas-ligand in bladder cancer in vitro and in vivo. *Urologic Oncology*.

[17] Srivastava A. K., Singh P. K., Singh D., Dalela D., Rath S. K., Bhatt M. L. B. (2014). Clinical utility of urinary soluble Fas in screening for bladder cancer. *Asia-Pacific Journal of Clinical Oncology*.

[18] Pinto Á., Redondo A., Zamora P., Castelo B., Espinosa E. (2010). Angiogenesis as a therapeutic target in urothelial carcinoma. *Anti-Cancer Drugs*.

